# Editorial: The distinct molecular and cardiometabolic characteristics of the soleus and slow oxidative muscle enhancing chronic disease prevention and healthy aging

**DOI:** 10.3389/fendo.2026.1911333

**Published:** 2026-07-17

**Authors:** Marc T. Hamilton, Sebastian Bohm, Vihang A. Narkar, Zhen Yan, Theodore W. Zderic

**Affiliations:** 1 Department of Health and Human Performance, University of Houston, Houston, TX, United States; 2 Department of Biology and Biochemistry, University of Houston, Houston, TX, United States; 3 Department of Training and Movement Sciences, Humboldt-Universität zu Berlin, Berlin, Germany; 4 Berlin School of Movement Science, Humboldt-Universität zu Berlin, Berlin, Germany; 5 Brown Foundation Institute of Molecular Medicine, McGovern Medical School, University of Texas (UT) Health, Houston, TX, United States; 6 The University of Texas MD Anderson Cancer Center University of Texas (UT) Health Graduate School of Biomedical Sciences, Houston, TX, United States; 7 Center for Exercise Medicine Research at Fralin Biomedical Research Institute at Virginia Tech Carilion, Roanoke, VA, United States; 8 Department of Human Nutrition, Foods, and Exercise, College of Agriculture and Life Sciences, Virginia Tech, Blacksburg, VA, United States

**Keywords:** calf muscle pump, Soleus Push Up (SPU), cell to cell communication (extracellular vesicles and myokines), postprandial glucose regulation, sedentary behavior replacement, postural balance, lower extremity venous hemodynamics, soleus muscle anatomy

## Introduction

More than a century before the advent of modern muscle physiology, Louis-Antoine Ranvier demonstrated that mammalian soleus muscle possesses an inherent phenotype optimal for endurance with its signature slow contraction speeds, high fatigue resistance, and tortuous microvasculature across wild hares, domesticated rabbits, and cats (1, 2). Currently, a momentum is building to understand how this highly conserved, slow-oxidative phenotype—alongside other unique anatomical and molecular properties—impacts cardiometabolic health and aging. Ultimately, this line of inquiry seeks to leverage the muscle’s specialized phenotypes to combat widespread chronic conditions, such as type 2 diabetes, while promoting healthy aging.

As referenced in the text below, the soleus has been described as a key “postural muscle”, a key cardiometabolic engine, and a potential systemic mediator of tissue cross-talk. Accumulating evidence highlights the soleus’s unique role in steady locomotion (3–5), perturbation recovery (6) and maintaining postural balance in the elderly (Fletcher & Strzalkowski), regulating blood pressure (Tremblay et al.), and facilitating fluid balance via lymphangiogenesis (Tamura et al.). From a metabolic and endocrine standpoint, targeting local soleus contractile activity to sustain its high oxidative metabolic rate is sufficient to improve systemic lipid utilization alongside postprandial glucose and insulin regulation (Hamilton et al.). Furthermore, emerging paradigms position the soleus as a specialized secretory organ, which releases a diverse array of humoral factors or myokines (7) and extracellular vesicles (EVs) that may act as untapped systemic signaling mediators for endocrinological investigation (8, 9). Within this context, this editorial frames how the soleus elicits distinct systemic benefits when called upon to work, particularly during periods of prolonged contractile activity.

Across all mammalian models studied, the soleus possesses distinct physiological attributes. Importantly, these highly specialized properties remain fully preserved in humans; even within a modern, increasingly sedentary lifestyle, the human soleus retains its evolutionary role in balance and ambulation (3) (Fletcher & Strzalkowski), alongside a massive latent potential for sustained oxidative metabolism (10). One major difference between humans and other mammals is the often underappreciated fact that the human soleus is very large relative to body weight. While the human soleus comprises approximately 1.3% of total body weight, it accounts for only ~0.1% of body weight in rodents (11); furthermore, it constitutes ~60% of total triceps surae mass in humans compared to less than 10% in rats and mice (12). This makes it more feasible that this muscle can exert systemic effects during acute contractions when its impact can be amplified. When mobilized via sustained activation, its unique structural and biochemical machinery serves as a primary lever for driving comprehensive systemic benefits (Hamilton et al.). While widely recognized for harboring the highest concentration of slow-twitch oxidative fibers, its specialized nature extends far beyond classic fiber-type categorization. Remarkably, transcriptomic mapping reveals that the global gene expression profile of the soleus aligns more closely with non-locomotor, highly active structures—such as the diaphragm, tongue, and extraocular muscles—than with its neighboring lower-leg counterparts (13).

In terms of future research related to endocrinology, rather than viewing the soleus purely as a model for slow-twitch contractions and postural stability, advancing human health and longevity may include how it communicates with other tissues via blood-borne secretory factors. There appears to be a contractile dependent and fiber type specific skeletal muscle communication with other tissues via the secretion of diverse proteins and other secretory factors (8, 9, 14). Given the soleus’s evolutionary design for frequent recruitment suggests it may have a specialized secretory capacity compared to muscles that contract only intermittently for brief periods of time (8, 9). The specialized molecular properties—combined with a rich microvasculature and lymphatic (15) network optimized for delivering proteins and cellular cargo to the systemic circulation—underpins the soleus’s potential capacity to operate as an endocrine organ that can be voluntarily regulated by contractile activity (8, 9). Crucially, while modern sedentary behaviors largely leave this tissue underutilized, the hypothesis that this secretory potential can be intentionally leveraged by replacing inactive sitting with targeted, prolonged local contractile activity opens exciting new doors for therapeutic intervention (10).

## The soleus is anatomically and biomechanically optimized to execute its necessary role in postural stability and efficient ambulation

The soleus has an outsized role in maintaining balance, preserving ambulatory function, and preventing falls in aging populations. Central to postural stability, the soleus is mechanically specialized for sustained, low-intensity plantarflexion force generation during upright stance (Fletcher & Strzalkowski).

The soleus is critical for reflexive postural control due to its low recruitment threshold motor units, tonic activation patterns, and high reflex gain. Its high density of muscle spindles relative to the smaller, neighboring gastrocnemius muscle (16) supports its evolutionary design as a reflexive stabilizer (Fletcher & Strzalkowski).

Targeted neuromuscular training represents a promising, yet underappreciated, strategy for optimizing postural control and mitigating fall risk in older adults (Fletcher & Strzalkowski).

## Concise listing of unique anatomical structure characteristics of the human soleus in comparison to other muscles and soleus in other mammals

- The evolutionary enlargement of the human soleus is a structural prerequisite for bipedalism, distinguishing human locomotion from quadrupedal mammalian transit (17). We believe that this expanded muscle volume may also carry unexpectedly profound implications for whole-body metabolic and hormonal regulation, as explained below.
- The human soleus possesses the greatest force-generating capacity of any lower limb muscle (e.g., gluteus maximus, vastus lateralis, gastrocnemius, and 27 muscles in total), a property driven by its high physiological cross-sectional area, exceptionally short muscle fibers, and highly pennated architecture (18).
- In humans, the soleus is unusually large relative to total body mass compared to other mammals, including non-human primates (11, 12, 17). Were this not the case, human postural stability/locomotor efficiency would be compromised and any possible systemic effects of local contractile activity by the human soleus would obviously be much less.
- The soleus inserts into the Achilles tendon — an extremely long and thick tendon — which allows the muscle to contract near-isometrically and economically during locomotion by storing elastic energy within the tendon matrix. This effectively minimizes the changes in the range of motion and velocity within the soleus fascicles in order to reduce the energy cost of these weight bearing activities during walking and running (3, 19, 20).
- The soleus across nearly all studied species is profoundly enriched with Type I slow-oxidative fibers (11, 21–23; reviewed in 12), ensuring the high economy required for human upright posture and bipedal ambulation.
- The soleus during Soleus Push Up (SPU) contractions (10) is by design the exact opposite of the high economy of the relatively isometric contractions of upright posture and ambulation (3); it is inefficient because it uses more fuel by requiring moderate to high velocity shortening (i.e., large range of motion, concentric) contractions (e.g., “The Fenn Effect”; 24, 25).

## Hemodynamic regulation

Tremblay et al. investigated the baroreflex-mediated activation of the lower leg muscles during upright standing. Their findings demonstrated that both prolonged (60-day) head-down tilt bed rest (HDBR) and biological aging significantly reduce muscle-pump baroreflex causality in the soleus and lateral gastrocnemius muscles. This specific reflex pathway plays a vital, necessary role in blood pressure regulation during orthostatic challenges, preventing venous pooling and subsequent orthostatic hypotension. The authors noted that their findings reveal shared phenotypic effects of aging and prolonged unweighting (HDBR) on the neurally mediated mechanisms governing cardiovascular stability during upright stance. They concluded that these data have direct implications for developing muscle-specific countermeasure strategies to mitigate the adverse cardiovascular and neuromuscular effects of spaceflight, prolonged clinical bed rest, and sedentary aging.

Tamura et al. demonstrated for the first time that voluntary wheel-running exercise increases lymphatic vessel density in skeletal muscle. Strikingly, this adaptation was isolated to the slow oxidative soleus muscle and was completely absent in the fast-twitch plantaris muscle. The authors noted that previous treadmill training protocols (26, 27) were not sufficient to elicit changes in skeletal muscle lymphatic density, likely due to a substantially lower training volume and duration (3–6 km/week compared to the 57 ± 12 km/week achieved via voluntary wheels). Notably, older mice—which failed to exhibit alterations in soleus lymphatic density—spontaneously ran less than half of this distance (Tamura et al.). This suggests that a high threshold of volume and duration is necessary to trigger lymphatic remodeling in slow-twitch tissue, a premise that future clinical and animal studies must confirm.

## Concise listing of features of the soleus for cardiovascular physiology

- Historical microvascular assessments by Ranvier demonstrated that the rabbit soleus exhibits an enriched, highly tortuous capillary network compared to fast-twitch muscles, even in sedentary animal models (1, 2); this unique architecture optimizes transit time and tissue oxygenation and extraction independently of prior exercise conditioning (28).
- The soleus exhibits a uniquely elevated blood flow response during contractions (29); for a given level of oxygen consumption, hyperemic blood flow is significantly higher in the soleus than in fast-twitch glycolytic muscles, ensuring superior matching between local O2 delivery and substrate utilization (30, 31).
- The Clinical Double-Edged Sword: The very unique vascular anatomy of the soleus is key to our and others’ viewpoints about the soleus (32, 33). Clinical evidence indicates that the expansive venous sinuses of the soleus are the primary origin site for most lower-extremity thrombi that subsequently migrate to cause pulmonary embolisms (34). While this unique structure is part of the human anatomy to make the soleus an incredibly powerful pump, it comes with a major physiological vulnerability. Because the large sinuses in the soleus are wide, thin-walled, valveless, and often compressed by tight internal septa, they are the number one site in the human body for blood stasis (pooling) during prolonged inactive sitting, standing, or bed rest (32). This anatomical uniqueness is exactly why deep vein thrombosis (DVT) so frequently originates inside the hidden sinuses of the soleus muscle. However, blood clotting in these vessels is not possible during periods of high blood flow during local soleus contractile activity, thereby raising the importance of a high duration of daily contractile activity for this serious and common condition, especially in the elderly.
- The human soleus is the peripheral heart. The venous network within the human soleus muscle is substantially *more developed quantitatively* and possesses highly *unique qualitative structural features* that set it apart from other skeletal muscles. The soleus muscle is structurally and functionally necessary to drive the calf muscle pump during ambulation, facilitating efficient venous return to the heart in tandem with exceptionally high baseline blood flow capacity (35). Because humans are obligate bipeds, we fight gravity to return blood from our lowest extremities to the heart. The human soleus has evolved into a highly specialized vascular organ, commonly nicknamed the “peripheral heart” or “second heart”.
- The large and venous rich soleus contains a reservoir of blood to increase cardiac stroke volume during muscle contractions. Instead of tiny vessels, the soleus contains large, dilated, sac-like venous cavities known as soleal sinuses. This single muscle acts as the primary reservoir for the lower leg, holding a substantial volume of stagnant or slow-moving blood when you are standing still, waiting to be forcefully ejected upon movement. Histologically, regular veins possess a distinct muscular wall layer (the tunica media) to maintain tone. The walls of the soleal sinuses are uniquely thin, composed almost entirely of endothelium and a delicate layer of connective tissue. They lack independent contractile strength because they do not need it; they rely entirely on the mechanical compression of the surrounding muscle fibers to collapse them.
- Immense Network Density: Medical dissections reveal that in most skeletal muscles, veins are small conduits (venae comitantes) that simply mirror the arterial supply to drain metabolic waste. In contrast, the soleus has numerous veins that do not run next to an artery. Therefore, contractile activity of the muscle is particularly important for the pumping of the blood flow to prevent perfusion insufficiency and a thrombus to form during bed rest or similar conditions. During dynamic contractile activity, the venous system in the soleus is structurally organized to create an effective fluid propulsion system for reducing hydrostatic pressure in the skin, feet, ankles and lower legs (a diastolic-like suction between contractions) followed by a systolic-like pumping during each concentric shortening contraction.
- The low systemic pressor response elicited by isolated soleus contractions in animals (36), is an effect driven in part by the lower sensitivity of soleus group III and IV afferent neurons in response to local metabolic changes (37). This mechanism is consistent with the observation that blood pressure and heart rate are maintained near resting with locally intense soleus contractions during SPU activity in humans (10).
- The smaller systemic homeostatic disturbance in blood pressure and heart rate is also consistent with a smaller sympathetic nervous system response in humans when contracting the soleus muscle—measured via muscle sympathetic nerve activity (MSNA)—when compared to equivalent contractile activity targeting the forearm or other leg muscle groups (38).

## Leveraging distinct soleus substrate kinetics: maximizing systemic lipid and glucose clearance by minimizing glycogenolysis

In metabolic physiology, proving that an intervention is sufficient to alter whole-body homeostasis represents an exceptionally high scientific bar. Proving sufficiency requires demonstrating that a single, isolated physiological event can independently drive systemic regulation without relying on broader systemic adaptations. Localized contractile activity of the human soleus meets this rigorous standard: it is entirely sufficient to markedly mitigate the postprandial glucose response following carbohydrate ingestion (10).

Even though the soleus is up to about 10 times larger in humans compared to lab rats (~1.3% of body weight in humans), it is still a small fraction of the muscle mass used in traditional “whole-body” exercises such as walking and cycling. Addressing whether the efficacy of this localized, low-mass contractile activity was comparable to traditional large-muscle exercises of shorter duration, Hamilton et al. demonstrated a profound physiological paradox. Isolated voluntary SPUs—even when at a level that elevates whole-body energy expenditure by a modest ~1 kcal/min—are sufficient to robustly accelerate glucose oxidation to a degree that attenuates postprandial blood glucose by 50 mg/dL at 1–2 hours post-ingestion (when hyperglycemia is most related to diverse kinds of pathological prognosis). This substantial magnitude of effect was recently replicated and confirmed by an independent laboratory (39). Crucially, the recent article highlighted physiological mechanisms for why conventional large-muscle exercise modalities are surprisingly ineffective at acutely suppressing postprandial glycemic excursions to this magnitude, and in some instances, may even adversely impact postprandial glycemia (Hamilton et al.).

## Concise listing of the unique metabolic features of soleus substrate utilization

**Figure 1:**
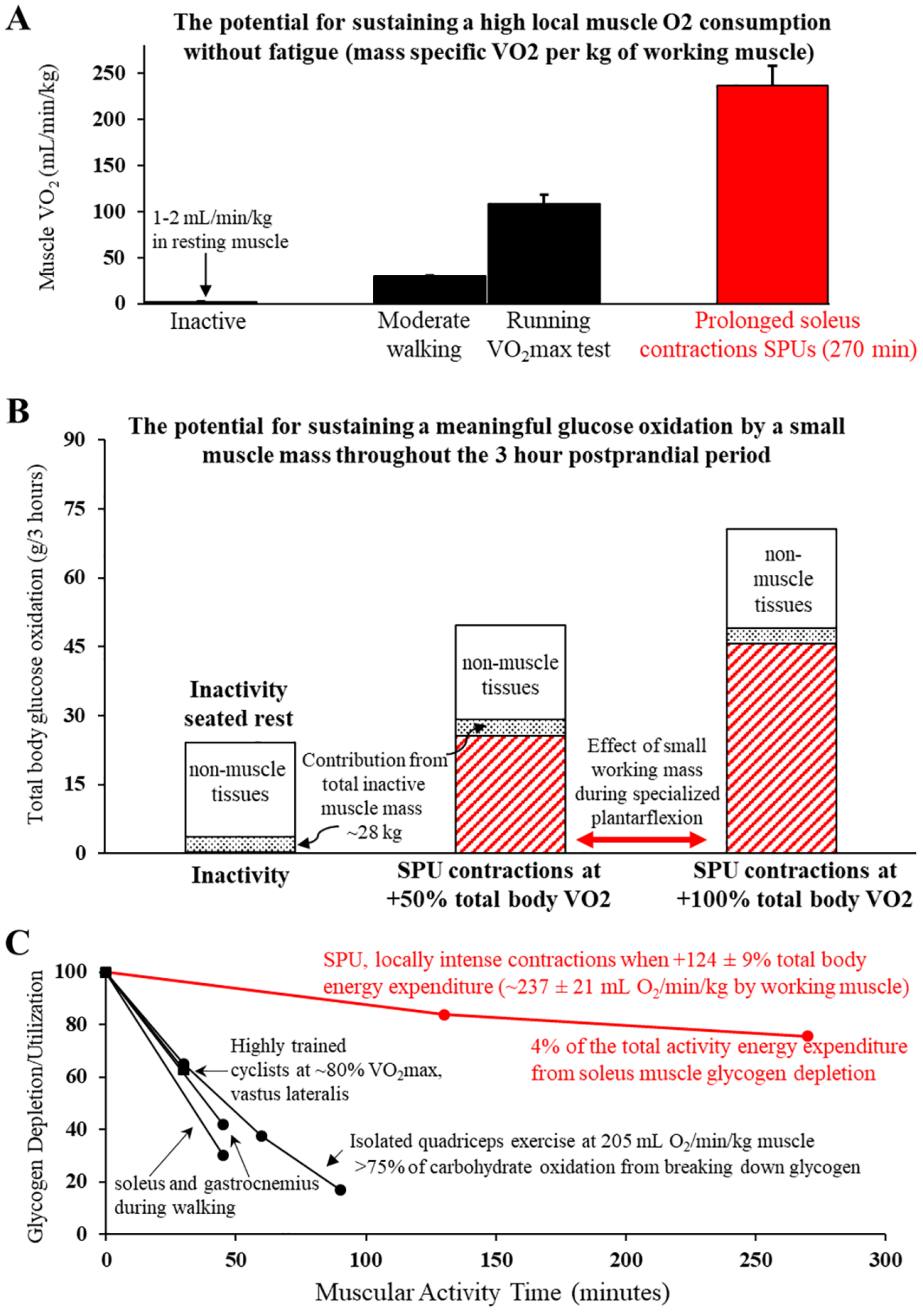
A unique type of locally intense, but fatigue-resistant contractile activity dominated by a slow-twitch oxidative muscle (**A**, Soleus Push Up or SPU) provides a method to amplify the influence of muscle metabolism on oxidative metabolism throughout the postprandial period **(B)**, with relatively less reliance on intramuscular glycogen than some other types of muscular activity **(C)**. **(A)** The SPU contractions can sustain a relatively high mass specific muscle VO2 compared to resting muscle and estimates of large muscle mass activities in the same volunteers during moderate walking and even running at VO2max (10). **(B)** The calculated contribution of skeletal muscle tissue to total body glucose oxidation is enhanced by 2 levels of SPU contractions. The moderate intensity SPU (~100% increase in total body VO2) and also the light intensity at half that level provided a meaningful process to continuously increase the contribution of skeletal muscle to total body glucose metabolism throughout the 3 hour postprandial OGTT. Note that while it is known skeletal muscle is not the dominant tissue for oxidative metabolism of blood glucose in the postprandial period  (40), this singular action of SPU contractions is sufficient for muscle to dominate the whole body responses. **(C)** In comparison to other types of muscular activity that also demand a high local oxygen demand, the SPU contractions were found to cause relatively little glycogen depletion in the soleus compared to the vastus lateralis during intense cycle ergometry (41) or isolated quadricep muscle contractions (42), and both the soleus and gastrocnemius during a much larger muscle mass activity like brisk uphill walking (43). Figure reproduced from Reference 10.

- During large muscle mass exercise (treadmill walking), the human soleus does break down muscle glycogen at a high rate like other muscles (43). Intramuscular glycogen is traditionally thought of as the dominant fuel source for most forms of moderate-to-vigorous intensity physical activity lasting one hour or less, during which circulating blood-borne substrates contribute less to muscle bioenergetics (Hamilton et al.). However, a new concept is that during specialized isolated contractile activity of the soleus (SPU contractions), it can minimize its reliance on glycogenolysis by shifting the metabolic demand toward blood-borne glucose and circulating lipids, thereby serving as a powerful systemic clearance mechanism for blood glucose and lipids (Hamilton et al.).
- Isolated soleus contractions performed at a high rate of local oxidative metabolism (SPU activity) use negligible muscle glycogen to sustain prolonged contractions (10). Even untrained men and women with a low VO_2_max use negligible muscle glycogen over 4.5 hours of soleus contractions at 237 mL O_2_/min/kg. In contrast, even highly trained endurance athletes when cycling at a similar local muscle VO2 (~235 mL O_2_/min/kg recruited muscle) use glycogen at a much higher rate (See **Figure 1**; Hamilton et al).
- The soleus muscle inherently has less glycogen phosphorylase (44–48) and glycogen debranching enzyme (47) which support the potential for less reliance on glycogen than other muscles under some hormonal conditions (e.g. low stress small muscle mass activity like SPUs that do not raise epinephrine).
- The soleus possesses ~10 times more capillary-bound lipoprotein lipase (LPL) compared to the fast-twitch skeletal muscles of rodents (49–53), maximizing its capacity to hydrolyze and clear circulating triglycerides.
- A higher percentage of Type I slow-twitch fibers is tightly correlated with superior lipid oxidation rates in humans during active contraction (54–57).
- This robust lipid-oxidizing capacity in Type I fibers is driven by a 2-to-3-fold higher expression of key lipolytic and catabolic proteins—including ATGL, HSL, Perilipin 2, Perilipin 5, BHAD, and LPL (49, 50, 55)—alongside a heightened ADP sensitivity of mitochondrial respiration when fueled by lipid-derived substrates (palmitoyl-l-carnitine + malate) rather than carbohydrate-derived substrates (pyruvate) (54).

## Conclusion

### Correctly harnessing sustained soleus activity: outsized influence on upright postural balance and sedentary cardiometabolic physiology

The human soleus possesses unique anatomical and physiological properties that enable it to perform a disproportionate volume of daily contractile work. Despite comprising only ~1% of total body mass, this single muscle exerts an outsized influence across two distinct behavioral states: upright posture and active sitting (SPU contractions). When standing, its specialized sensory and mechanical features provide the necessary stability to maintain human balance as a tall, upright pillar and walk with exceptional metabolic efficiency. In a striking functional divergence, when sitting, correctly targeting this muscle with a singular unloaded movement is sufficient to drive whole-body oxidative glucose metabolism to levels up to ~300% of resting metabolic rate, outstripping all other tissues combined (10). While current evidence confirms that sustained soleus activation is sufficient to optimize postprandial glucose and insulin regulation, these metabolic effects likely represent just the tip of the endocrinological iceberg. Ultimately, elucidating how the soleus functions as a potent secretory organ driving inter-organ cross-talk may reveal much more diverse systemic benefits mediated by the ability of the soleus to sustain fatigue-resistant contractile activity.
